# Epidemiology of SARS-CoV-2 Infection in Patients with Neuromuscular Disease and Inborn Errors of Metabolism: A Cross-sectional Study for a Pediatric Outpatient Referral in Japan

**DOI:** 10.24546/0100492945

**Published:** 2025-02-03

**Authors:** SUNGWON HONG, KIIKO IKETANI, SHOKO SONEHARA, HIROAKI HANAFUSA, YOSHINORI NAMBU, KANDAI NOZU, HIROYUKI AWANO, RYOSUKE BO

**Affiliations:** 1 Department of Pediatrics, Kobe University Graduate School of Medicine, Kobe, Japan; 2 Department of Endocrinology and Metabolism, Hyogo Prefectural Kobe Children’s Hospital, Kobe, Japan; 3 Organization for Research Initiative and Promotion, Tottori University, Yonago, Japan

**Keywords:** COVID-19, Neuromuscular diseases, Inborn errors of metabolism, Pediatric

## Abstract

The coronavirus disease 2019 (COVID-19) pandemic has affected people worldwide, and pediatric patients with underlying diseases are at high risk of developing severe COVID-19. However, there are limited reports on the clinical impact of COVID-19, especially in patients with underlying neuromuscular diseases (NMD) and inborn errors of metabolism (IEM). This study aimed to investigate the incidence and clinical presentation of COVID-19 in patients with NMD and IEM. This was a single-center, cross-sectional study of patients with NMD and IEM in Japan for 2 years, from April 1, 2020 to March 31, 2022. Among 255 participants with a median age of 14 (range: 0–50) years, 192 (75%) and 63 (25%) had NMD and IEM, respectively. Among 255 patients, 8 (5 NMD and 3 IEM) were positive for the anti-severe acute respiratory syndrome coronavirus 2 nucleocapsid antibody, and the incidence was considered 3%. All positive patients had mild or asymptomatic COVID-19. None of the patients exhibited moderate or severe symptoms. In conclusion, this study revealed that the incidence of COVID-19 was low, and mild or subclinical infection was common even in patients with NMD and IEM, who may be at a higher risk of severe COVID-19.

## INTRODUCTION

The coronavirus disease 2019 (COVID-19) pandemic, which began in December 2019, significantly affected the world. On March 13, 2020, Japan declared a state of emergency based on the Law on Special Measures against COVID-19, which caused great concern, especially for children with underlying diseases and their caregivers. On May 4, 2023, based on the development of vaccines and treatments, the World Health Organization declared the end of the Public Health Emergency of International Concern [1].

In Japan, the Infectious Diseases Control Law classifies infectious diseases 1–5 based on their infectiousness and severity. Although COVID-19 was equivalent to Class 2, it was reclassified as Class 5 on May 8, 2023 [2]. However, regardless of the reclassification, COVID-19 continues to be a threat to human health. In addition, the incidence of COVID-19 is expected to increase as people begin to lift their restrictions regarding social activities.

In neuromuscular diseases (NMDs), features that have a high risk for developing severe COVID-19 include muscular weakness of the chest or diaphragm, kyphoscoliosis, use of ventilation via mask or tracheotomy, weak cough and airway clearance, and presence of tracheostoma [3]. In inborn errors of metabolism (IEM), the accumulation of toxic substances and decreased energy production during fever, vomiting, diarrhea, and other symptoms associated with infectious diseases can lead to an imbalance in the body, resulting in metabolic decompensation [4]. Therefore, COVID-19 increases the risk of developing severe conditions in patients with underlying diseases [5].

However, there are limited reports on the clinical impact of COVID-19 in patients with underlying NMDs and IEM, whose approximate prevalence ranges from one in thousands to hundreds of thousands [4, 6, 7]. Although a systematic review using the European Registry and Network for Intoxication Type Metabolic Diseases Consortium database evaluated the COVID-19 outcomes for organic acid metabolism and urea cycle abnormalities [8], the COVID-19 case reports remain scarce for other types of IEM. Moreover, the clinical data are scarce, and the risk of infection in patients with NMD or IEM is not fully understood.

In this study, we investigated the incidence and clinical features of COVID-19 among patients with NMDs and IEM who visited the Department of Pediatrics at Kobe University Hospital during the COVID-19 pandemic.

## MATERIALS AND METHODS

### Study design and patients

This cross-sectional study included patients who met all the following criteria: 1) visited the Department of Pediatrics, Kobe University Hospital once or more in the 2-year period from April 1, 2020, to March 31, 2022; 2) had a confirmed diagnosis of NMD or IEM; and 3) had access to residual serum samples.

### Samples and SARS-CoV-2 antibody test

The residual serum samples were first stored at 4–5°C in the refrigerator, and within 7 days, they were moved to store at −20°C or below. The samples were sent to SRL, Inc. (Tokyo, Japan) within 3 months for SARS-CoV-2 antibody testing. The test kit was Elecsys® Anti-SARS-CoV-2 (RUO) (Roche Diagnostics K.K., Tokyo, Japan).

### Definition and classification of infectious patients with COVID-19

The patients who tested positive with Elecsys® Anti-SARS-CoV-2 (RUO) were defined as infected. Elecsys® Anti-SARS-CoV-2 (RUO) is a qualitative antibody test for mature antibodies (anti-nucleocapsid [N] antibodies) against SARS-CoV-2 using the ECLIA method [9]. If a patient is infected with COVID-19, both N and neutralizing antibodies (anti-spike [S] antibodies) are present [10]. On the other hand, the vaccine recipients are positive only for S antibodies and negative for N antibodies [10, 11]. Thus, a positive result by Elecsys® Anti-SARS-CoV-2 (RUO) meant a history of natural infection with COVID-19.

The infected patients were classified into three groups according to the presence or absence of symptoms and time of appearance as follows: confirmed symptomatic patients, patients with a confirmed diagnosis by antigen test or polymerase chain reaction (PCR) at the time of symptom onset; suspected symptomatic patients, patients with COVID-19 related symptoms within 1 year from the time of antibody positivity but without confirmed COVID-19 antigen test or PCR at the time of symptom onset; subclinically infected patients, patients with no COVID-19 related symptoms within 1 year from the time of antibody positivity.

The symptom severity was classified based on “COVID-19 Treatment Guide Version 10.1 [5]. The Ministry of Health, Labour and Welfare of Japan published as follows: mild, no respiratory symptoms or having only cough without dyspnea; moderate, dyspnea or signs of pneumonia; severe, hospitalized in an intensive care unit or on artificial respiration.

### Clinical characteristics

The patients’ clinical data were obtained from their electronic medical records. Data regarding sex, underlying disease, and age at the time of blood collection were obtained. For anti-SARS-CoV-2 antibody-positive patients, the following additional information was collected: presence of respiratory support, treatment of the primary disease, social history, time of antibody positivity, COVID-19 classification (severity), time of appearance of COVID-19 symptoms, and vaccination history up to the time of antibody positivity.

### Ethics

This study was approved by the Ethics Review Committee of Kobe University Hospital (approval number: B2056710). About informed consent, the patients had the right to opt out of the study on the website. Patients who refused to participate were excluded.

## RESULTS

### Background of patients

A total of 255 individuals, 213 men (84%) and 42 women (16%), participated in this study. The median age at participation was 14 (0–50) years. Regarding underlying diseases, 192 (75%) patients had NMDs, and 63 (25%) had IEM (Tables I-1 and I-2). Duchenne muscular dystrophy (DMD) was the most common NMD, followed by Becker muscular dystrophy (BMD). Hepatic glycogen storage diseases were the most common type of IEM.

### Clinical characteristics of COVID-19

Of the 255 patients, 8 (3%) were infected. Five (3%, 5/192) patients had NMD, and 3 (5%, 3/63) had IEM. Of the eight infected patients, 2, 2, and 4 patients were “confirmed symptomatic,” “suspected symptomatic,” and “subclinically infected,” respectively. All confirmed symptomatic and suspected symptomatic patients were classified as “mild,” and none were “moderate” or “severe.” The clinical data of anti-SARS-CoV-2 antibody-positive patients are shown in Table II.

Of the five positive patients with muscular dystrophy, one was “confirmed to be mildly symptomatic,” and the other was “suspected to be symptomatic” case with mild symptoms. The remaining three patients were “subclinically infected.” Patient 3, who required assistance from a wheelchair, has already been administered lisinopril and carvedilol for cardiac dysfunction by primary disease. No ventilator, such as noninvasive positive pressure ventilation, was used. Owing to the patient’s primary disease, there was concern regarding the risk of severe COVID-19. Thus, he was admitted to the hospital as a precaution; however, his subsequent progress was good and did not require additional treatment. Even though none of the patients were vaccinated, they were not severely ill.

Regarding IEM, one patient had citrin deficiency, one had phenylketonuria, and one had argininosuccinic aciduria. The patient with citrin deficiency was “suspected to be symptomatic” case with mild symptoms such as a slight fever for a few days and abnormal taste for one day. The patient with phenylketonuria was “subclinically infected.” Patient 8 with argininosuccinic aciduria was “confirmed mildly symptomatic,” with fever, vomiting, and coughing but no increase in ammonia levels. This patient was vaccinated in July 2021 and August 2021.

## DISCUSSION

In this study, 3% (8/255) of participants tested positive for COVID-19. All eight positive patients were either confirmed or suspected to be mildly symptomatic or subclinically infected. There were no patients with “moderate” or “severe” symptoms.

In a retrospective case-control study of 71,656 patients under 18 years of age with chronic complex neurological disorders [12], Chi et al. reported that patients with muscular dystrophy and myopathy were at a higher risk of severe COVID-19 (odds ratio = 3.22; 95% confidence interval [CI], 2.73–3.84). However, their study had two limitations. First, the severity of COVID-19 was classified as moderate or severe if oxygen administration was required; however, this study failed to distinguish patients receiving chronic respiratory support. Second, this study may not have included individuals from outpatients with mild illnesses. Levine et al. reported the incidence of COVID-19 in 116 patients with Duchenne/Becker muscular dystrophy who were treated at a single center in Israel from March to December 2020 [13]. The incidence rate of COVID-19 in this study was 6% (7/116), with six patients with DMD and one with BMD. Two of these patients were symptomatic and required hospitalization, and both patients were managed with cough assistance and discharged after 5 days. In contrast, the remaining five (71%) positive patients were subclinically infected. Benito et al. also reported the clinical presentation of 29 pediatric NMD patients with COVID-19, including four patients with DMD, one with BMD, one with limb-girdle muscular dystrophy, and 11 with spinal muscular atrophy (SMA). Three patients (10%) with SMA type 1, presenting with moderate COVID-19, were hospitalized for an average of 7 (range: 3–10) days. In contrast, the remaining 89% of the patients were clinically categorized as asymptomatic or mild cases [14]. While patients with underlying NMD are considered to be at high risk of severe COVID-19, this study and previous reports suggest that the proportion of patients with subclinical infection or mild disease may also be sufficiently high.

Regarding IEM, one patient with phenylketonuria had a subclinical infection, and one patient with citrin deficiency was suspected as mildly symptomatic. The patient with urea cycle disorder was confirmed to be mildly symptomatic. Regarding IEM and COVID-19, Kahraman et al. studied the risk of COVID-19 in 1,841 patients with inherited IEM, followed at a single referral center [15]. Of the 131 pediatric IEM patients infected with COVID-19, 8 (6%) had moderate or severe disease, and 123 (94%) were mild or asymptomatic. Of the 92 adult IEM patients with COVID-19, 6 (7%) had moderate or severe disease, and 86 (93%) were mild or asymptomatic. Mütze et al. also reported the systematic disease course and outcome of SARS-CoV-2 infection in patients with organic acidurias and urea cycle disorders, which are at high risk of rapid deterioration triggered by infection [8]. In this review, 49 of 59 (83%) infected individuals were treated at home and showed no or mild clinical symptoms. The remaining 10 patients were admitted to a hospital for vomiting, reduced oral intake, pneumonia, and so on. None of the patients required a multidisciplinary treatment in the ICU. All patients (100%) were managed at home or in the outpatient department, and most hospitalized patients (90%) showed complete recovery. COVID-19-related metabolic decompensations were usually mild, without an increased risk of ICU treatment. In contrast, Zubarioglu et al. reported the clinical course of COVID-19 in 11 patients with a diagnosis of intoxication type of IEM. The patients did not have any severe disease; however, two patients diagnosed with propionic acidemia, one with methylmalonic acidemia, and one with 3-hydroxy-3-methylglutaryl-CoA lyase deficiency presented with clinical and biochemical findings of an acute metabolic attack [4]. In this study, although the number of reported cases was small, all patients were considered to have mild or asymptomatic COVID-19. Argininosuccinic aciduria is known to cause metabolic failure with hyperammonemia, which is initially triggered by infection. However, there was no increase in ammonia levels of the patient in this study. Similar to patients with NMD, most patients with IEM were likely to have mild or subclinical COVID-19.

In the present study, there were no cases of severe COVID-19 in either the NMD or IEM group. Respiratory dysfunction, such as weak cough and weak respiratory muscles seen in NMD, including SMA and DMD, is a significant risk factor for COVID-19 severity. Innate immunity and acquired immunity are also essential factors for COVID-19 severity [16]. Taking immunosuppressants is a risk factor for severe COVID-19 [5]. Although DMD is not usually reported to cause immune dysfunction, steroids may be taken to alleviate progressive muscular weakness in some cases. In this study, none of the patients with DMD who tested positive for COVID-19 were taking steroids. If they had been taking steroids, it is undeniable that the COVID-19 could have been severe. Immunological abnormalities have been reported in some IEMs, including propionic acidemia (PA) and methylmalonic acidemia (MMA) [17]. Since this study did not detect the patients with MMA and PA who had COVID-19, it is difficult to further discuss about whether patients with IEMs related to congenital immunodeficiency tend to have severe COVID-19. While no reports about the risk for immunodeficiency have been reported for citrin deficiency and argininosuccinic aciduria, two reports have been reported on phenylketonuria, which suggested patients with phenylketonuria might have lower IgG or IgA than healthy individuals [18, 19]. However, there were no reports of patients with phenylketonuria being immunologically vulnerable and prone to severe viral infections. More accumulation of cases is necessary to explore the association between immunological abnormalities associated with IEM and the severity of COVID-19.

The strength of this study is that it reported the incidence and clinical course of COVID-19 in patients with rare underlying diseases, whose prevalence is one in thousands to hundreds of thousands. The most frequent hereditary muscle disease, DMD, has an incidence of 4.8 per 100,000 people [20]. Since the incidence of IEM is usually one in tens to hundreds of thousands and there have been few comprehensive reports on COVID-19 in Japan for these rare diseases, this study could be a critical report. Furthermore, this study was conducted in patients who regularly visited the outpatient clinic and whose blood samples could be obtained. Therefore, subclinical infections could be accurately assessed. The severity classification of positive cases may reflect the epidemiological data of patients more accurately than if only symptomatic cases were collected. Moreover, many patients with NMD or IEM and their families are very concerned about the incidence of COVID-19. Despite the lack of sufficient evidence, the study results show that many patients with NMD and IEM have mild or asymptomatic infection, which may reassure them.

The limitations of this study include the fact that it was a single-center study that was limited to cases for which blood samples were available. Since not all patients with NMD or IEM in our center were tested, the data may not accurately represent the number of infected people over the entire period. Another limitation was the small sample size due to the rarity of the underlying diseases. Furthermore, data on clinical symptoms were mainly obtained from the patients or their family members. This might have resulted in inadequate information.

We analyzed anti-SARS-CoV-2 antibody-positive cases in many patients with NMD and IEM at our hospital. The incidence of COVID-19 among patients with NMD and IEM was 3% from April 2020 to March 2022. All the eight positive cases were mild or asymptomatic. These results indicate that subclinical infection is common, even in patients with NMD and IEM, who may be at a higher risk of severe COVID-19.

## Figures and Tables

**Table I-1:** Disease background of 192 patients with NMD

Diagnosis	Number of patients
Duchenne muscular dystrophy (DMD)	91
Becker muscular dystrophy	64
Spinal muscular atrophy	14
Limb-girdle muscular dystrophy	9
Congenital myopathy	3
Congenital muscular dystrophy	2
Fukuyama congenital muscular dystrophy	2
Congenital myotonic dystrophy	2
Distal myopathy	1
Facioscapulohumeral muscular dystrophy	1
Symptomatic female carrier of DMD	1
Dermatomyositis	1
Polymyositis	1

**Table I-2:** Disease background of 63 patients with IEM

Diagnosis	Number of patients
Hepatic glycogen storage disease	10
Phenylketonuria	7
Citrin deficiency	7
Methylmalonic acidemia	6
Medium-chain acyl-CoA dehydrogenase deficiency	4
Propionic acidemia	3
Menkes disease	3
3-Methylcrotonyl-CoA carboxylase deficiency	2
Carnitine palmitoyltransferase II deficiency	2
Very long-chain acyl-CoA dehydrogenase deficiency	2
Argininosuccinic aciduria	2
Ornithine transcarbamylase deficiency	2
Homocystinuria	1
Holocarboxylase synthetase deficiency	1
Carbamoylphosphate synthetase 1 deficiency	1
Methionine adenosyltransferase deficiency	1
Pyruvate dehydrogenase complex deficiency	1
HMG-CoA synthase deficiency	1
Isovaleric acidemia	1
Primary carnitine deficiency	1
N-acetylglutamate synthase deficiency	1
Tay-Sachs disease	1
Galactosialidosis	1
Mucolipidosis	1
Mitochondrial disease	1

**Table II:** Clinical characteristics of eight patients with COVID-19

Patient No.	Diagnosis	Age	Sex	Respiratory support	Treatment for underlying disease	Social history	Time of antibody positivity	Clinical severity of COVID-19	Time of appearance of COVID-19 related symptoms	Status of vaccination before antibody positivity
**NMD**										
1	BMD	18	M	No	No medication	Student	2020/8	subclinical	None	None
2	LGMD	12	F	No	No medication	Student	2021/2	subclinical	None	None
3	DMD	23	M	No	Lisinopril Carvedilol	Support center for disabled people’s employment	2021/3	c. symptomatic (mild)	2021/1	None
4	LGMD	23	M	No	No medication	Working	2021/8	subclinical	None	None
5	DMD	9	M	No	No medication	Student	2022/3	s. symptomatic (mild)	2021/11 2022/2	None
**IEM**										
6	Citrin deficiency	12	M	No	Carbohydrates restricted food	Student	2020/12	s. symptomatic (mild)	2020/2	None
7	PKU	20	F	No	Phenylalanine restricted food	Student	2021/8	subclinical	None	None
8	ASA	21	F	No	Arginine, Levocarnitine, SPB	Unknown	2022/2	c. symptomatic (mild)	2022/1	Twice

NMD, neuromuscular disorders; IEM, inborn errors of metabolic disorders; BMD, Becker muscular dystrophy; DMD, Duchenne muscular dystrophy; LGMD, limb-girdle muscular dystrophy; PKU, phenylketonuria; ASA, argininosuccinic aciduria; SPB, sodium phenylbutyrate; c. symptomatic, confirmed symptomatic; s. symptomatic, suspected symptomatic.
